# NMDA receptor dependent anti-diabetic effects

**DOI:** 10.1186/2194-7791-1-S1-A26

**Published:** 2014-09-11

**Authors:** Jan Marquard, Silke Otter, Alena Welters, Diran Herebian, Fatih Demir, Annett Schroeter, Olaf Kletke, Martin Kragl, Daniel Eberhard, Barbara Bartosinska, Masa Skelin Klemen, Andraz Stozer, Martin Köhler, Alin Stirban, Freimut Schliess, Tim Heise, Stephan Wnendt, Marjan Slak Rupnik, Per-Olof Berggren, Nikolaj Klöcker, Thomas Meissner, Ertan Mayatepek, Eckhard Lammert

**Affiliations:** Department of General Pediatrics, Neonatology and Pediatric Cardiology, University Children's Hospital Düsseldorf, 40225 Düsseldorf, Germany; Institute of Metabolic Physiology, Heinrich-Heine University, 40225 Düsseldorf, Germany; Institute for Beta Cell Biology, German Diabetes Center (DDZ), 40225 Düsseldorf, Germany; German Center for Diabetes Research (DZD e.V.), 85764 Neuherberg, Germany; Institute of Neuro- and Sensory Physiology, Heinrich-Heine University, 40225 Düsseldorf, Germany; Institute of Physiology,Faculty of Medicine, University of Maribor, 2000 Maribor, Slovenia; The Rolf Luft Research Center for Diabetes and Endocrinology, Karolinska Institutet, 17176 Stockholm, Sweden; Profil Institute for Clinical Research, 41460 Neuss, Germany; MLM Medical Labs GmbH, 41066 Moenchengladbach, Germany

## Aims

In the central nervous system, NMDA receptors play a pivotal role, however, their role in pancreatic islets has been largely unexplored or is controversial. We hypothesized that NMDA receptors are involved in glucose stimulated insulin secretion from beta cells and might serve as novel drug targets for diabetes treatment.

## Methods

We generated mice with a pancreas-specific deletion of all NMDA receptors. The phenotype of these mice and their pancreatic islets were characterized in terms of insulin secretion, glucose tolerance and calcium oscillations. We also pharmacologically inhibited the NMDA receptors *in vitro* and *in vivo*. Subsequently, insulin secretion from isolated mouse and human islets as well as blood glucose and plasma insulin concentrations were analyzed in mice and via a clinical trial in type 2 diabetic patients.

## Results

Functional NMDA receptors are expressed in insulin producing cells. A pancreas specific NMDA receptor knockout selectively increases glucose stimulated insulin secretion *in vitro* and improves glucose tolerance *in vivo*. The pharmacological inhibition of NMDA receptors selectively increases glucose stimulated insulin secretion *in vitro* and improves glucose tolerance in C57Bl/6 and diabetic mice. In addition, we performed a clinical trial in patients with type 2 diabetes and provided evidence that the NMDA receptor antagonist Dextromethorphan (DXM) lowers blood glucose levels and increases insulin secretion without the risk of hypoglycemia.

## Conclusion

We show for the first time that NMDA receptors can be targeted genetically and pharmacologically in mice and men to selectively increase glucose stimulated insulin secretion with improvement of glucose tolerance. We uncovered a new role for NMDA receptors in the pancreas and showed that inhibiting these receptors might serve as a useful treatment of human diabetes in pediatric and adult patients.

